# SARS-CoV-2 drives upregulation of EpCAM in respiratory epithelial cells with hypercellular profile during severe COVID-19

**DOI:** 10.1007/s00011-026-02244-3

**Published:** 2026-06-20

**Authors:** Franklin Pereira Araújo, Geovane Marques-Ferreira, Camila Pacheco Silveira Martins da Mata, Erik Vinicius de Sousa Reis, Laura Cardoso Corrêa-Dias, Ágata Lopes-Ribeiro, Caio Wilker-Teixeira, Gabriel Eduardo Ribeiro Mendes, Vanessa Peruhype-Magalhães, Ana Carolina Campi-Azevedo, Olindo Assis Martins-Filho, Andréa Teixeira-Carvalho, Jordana Grazziela Alves Coelho-dos-Reis

**Affiliations:** 1 ProsPeRA Research Group. Laboratório de Virologia Básica E Aplicada, Departamento de Microbiologia, Instituto de Ciências Biológicas, Universidade Federal de Minas Gerais, Avenida Antônio Carlos, 6627 – Pampulha, Belo Horizonte, Minas Gerais 31270-901 Brazil; 2 Instituto René Rachou, Fundação Oswaldo Cruz (FIOCRUZ-Minas), Avenida Augusto de Lima, 1715, Barro Preto, Belo Horizonte, Minas Gerais 30190-002 Brazil; 3 Hospital Risoleta Tolentino Neves, Universidade Federal de Minas Gerais, Belo Horizonte, Minas Gerais Brazil

**Keywords:** Severe COVID-19, Epithelial cells, Cytokeratin, EpCAM, ICAM-1

## Abstract

**Background:**

At the end of 2019, severe acute respiratory syndrome (SARS) caused by the novel coronavirus SARS-CoV-2 emerged in China, representing one of the most significant global health threats of the twenty-first century. This study aimed to evaluate the phenotypic and cellular activation of epithelial cells in tracheal aspirate samples from patients with severe COVID-19.

**Methods:**

A total of 48 samples were collected from 28 mechanically ventilated patients and analyzed using flow cytometry and conventional microscopy.

**Results:**

The results revealed a high prevalence of basal, ciliary, undifferentiated cells, and pneumocytes displaying morphological abnormalities. Samples were classified into hypocellular and hypercellular profiles based on total cell counts. These profiles were associated with clinical outcomes, with hypercellular samples more frequently observed in patients who died. Hypercellular samples exhibited increased frequencies of basal, undifferentiated cells and pneumocytes, as well as a greater presence of CD45⁺ cells with elevated HLA-DR expression, indicating an influx of activated leukocytes and antigen-presenting cells. In contrast, hypocellular samples showed a higher proportion of CD45⁻PanCK⁺ cells, reflecting a predominance of epithelial cells. Hypercellular samples also demonstrated increased EpCAM⁺PanCK⁺ epithelial cells with more expression of Spike in the context of intense inflammation. ICAM-1 expression was elevated in CD45⁺ cells from hypercellular samples, while reduced in CD45⁻ cells. Finally, a superior connectivity was found for networks of correlations of hypercellular samples, especially amongst the compartments of cells expressing EpCAM.

**Conclusion:**

Together, these findings identify two distinct cellular profiles that may serve as prognostic indicators in severe COVID-19.

**Supplementary Information:**

The online version contains supplementary material available at 10.1007/s00011-026-02244-3.

## Introduction

In December 2019, a new virus named SARS-CoV-2 was identified after being isolated from bronchoalveolar lavage fluid samples of patients with severe pneumonia, later termed COVID-19 [1]. SARS-CoV-2 belongs to the species *Betacoronavirus pandemicum*, within the *Coronaviridae* family and *Orthocoronavirinae* subfamily. It is classified under the genus *Betacoronavirus*, as defined by the International Committee on Taxonomy of Viruses [2].

At the onset of infection, the virus primarily targets bronchoalveolar epithelial cells, vascular endothelial cells, and lung macrophages [3]. As the infection progresses, recruited macrophages and monocytes release cytokines that activate the adaptive immune response, including T and B cells. In severe cases [4, 5], as the disease progresses, the body triggers a localized inflammatory response, accompanied by the release of pro-inflammatory cytokines and chemokines, such as IL-6, IFN-γ, MCP-1 (CCL2), and IP-10 (CXCL10), into the bloodstream [5]. Lymphopenia, a hallmark of COVID-19, results from the migration of lymphocytes into the airways, accompanied by the infiltration of both lymphocytes and neutrophils [6, 7].

The bronchoalveolar epithelium serves as a primary physical barrier against microorganisms and is the first target of SARS-CoV-2. Within the epithelial compartment, several protective layers exist, starting with the mucus layer, followed by the ciliated epithelium [8]. Additionally, various cells with distinct morphologies are found attached to the lamina propria of the basal membrane of the respiratory epithelium. These include type I and II alveolar cells (AT1 and AT2), as well as basal, ciliated, clavate/club, goblet, and mucous cells [9].

Despite the importance of the respiratory tract, limited evidence exists on the phenotypic and functional characteristics of epithelial cells during severe COVID-19. It is known that SARS-CoV-2 causes remodeling of the bronchoalveolar epithelium to enhance infection and triggers airway inflammation, which leads to the cytokine storm [10, 11].

Therefore, this study aimed at assessing the phenotypic and cellular activation of tracheal aspirate cells from COVID-19 patients under mechanical ventilation in the intensive care unit, focusing on their association with morbidity and mortality. Investigating these cellular characteristics may provide valuable insights for treating and preventing severe cases of the disease, especially in the context of emerging SARS-CoV-2 variants.

## Material and methods

### Study population

Tracheal aspirate (TA) samples were collected in the morning from 28 patients under mechanical ventilation after their admission to Intensive Care Unit (ICU) of Risoleta Tolentino Neves Hospital (HRTN). The range of age of the patients is from 29 to 84. While all patients had at least one TA collected, a second sample was obtained 7 days after the first, from 14 patients, resulting in two time-point collections for this subgroup. The collections were performed using a bronchial secretion collector, resulting in a total of 48 samples. Patient demographics are presented in Supplementary Table 1. Comorbidity data were available for a subset of patients (n = 17). Among these, the most frequent comorbidities were hypertension (64.7%), diabetes mellitus (47.1%), and obesity (41.2%), followed by previous myocardial infarction (11.8%), hypothyroidism (11.8%), dyslipidemia (5.9%), chronic kidney disease requiring dialysis (5.9%), chronic obstructive pulmonary disease (5.9%), HIV infection (5.9%), glaucoma (5.9%), and Sjögren’s syndrome (5.9%). Percentages were calculated based on patients with available clinical information (Table 1). Inclusion criteria consisted of mechanical ventilation requirement and laboratory confirmation of SARS-CoV-2 infection. Comorbidities were not used as stratification variables for outcomes in this study. The samples were then processed and stored at − 80 °C for subsequent analysis. This study is prospective, exploratory, observational, and descriptive, and it was approved by the National Research Ethics Committee (CONEP) under the Certificate of Ethical Appreciation (CAAE) number 30846920.7.0000.0008. The Informed Consent Form (ICF) was signed by the patients’ guardians at the time of their inclusion in the study.

**Table 1:** Demographic and clinical characteristics of the COVID-19 group

Characteristics	COVID-19 patients
RT-PCR (%)	100%
Age	61 years (median)
Gender (F/M)	F—32%; M 68%
Comorbities	Obesity, hypertension, diabetes mellitus, smoking
Chest CT scan	Ground-glass opacity with bilateral involvement of 50% of the lungs
Mechanic ventilation (%)	100%
Gasometry	
SpO_2_ < 93%	34.8 and 249%
IRPM > 28	22 and 124
pCO_2_ > 50 mmHg	15 and 94,9
pH < 7.25	7.22 and 7.57
Outcome n (%)	
Death	20 (71.42%)
Discharge	8 (28.57%)

### Microscopic evaluation of pulmonary epithelial cells

To evaluate the microscopic profile of epithelial cells in the tracheal aspirate (TA), 50 µL of TA was pipetted onto one end of a glass slide. A second slide, held at a 45° angle, was used to spread the sample by moving it backward until it touched the drop and then forward to create a uniform smear. The slides were allowed to dry completely at room temperature with the samples facing upwards. Once dried, the slides underwent Panoptic rapid staining by sequentially submerging them in three solutions: triarylmethane, xanthene (red dye), and purple thiazine, each for 3 s, followed by gentle dipping and draining (Laborclin, Paraná, Brazil). After staining, the slides were rinsed with distilled water to remove excess dye and left to air dry. Finally, the dried slides were examined under a binocular microscope at 100 × magnification using immersion oil. The epithelial cells were counted using a method similar to a differential leukocyte count, with a total of 100 cells analyzed per slide. Photographs were taken and organized to emphasize key features, such as ciliated epithelial cells (both with and without cilia), basal cells with clustered nuclei, undifferentiated cells with prominent nuclei and cytoplasm, pneumocytes characterized by flat nuclei and vacuoles, and the presence of leukocyte infiltrates.

### Cell count

To evaluate the cellular profile of the pulmonary epithelium, 2–4 mL of tracheal aspirate was treated with 1% DTT and incubated for 15 min. The samples were then washed with 5 mL of 1 × PBS and filtered through a Cell Strainer attached to a 50 mL Falcon tube. Subsequently, the samples were centrifuged at 2,500 rpm for 10 min at 18 °C, and the supernatant was discarded. The resulting pellet was resuspended in 1 mL of 1 × PBS. For cell counting, 10 µL of the cell suspension was stained with trypan blue and placed in the counting chamber for analysis using the Invitrogen Countess 3 automatic cell counter (California, USA), in accordance with the manufacturer’s instructions. The total number of cells were recorded, followed by the calculation of a median value based on the live cell count per mL. Cellularity of tracheal aspirate samples was assessed by determining total and viable cell counts using Countess™ automated cell counting device (ThermoFisher Scientific, USA) as described by the manufacturer.

### Evaluation of phenotypic and cellular activation of epithelial cells present in the tracheal aspirate samples from severe COVID patients

After counting, the volume of remaining cells was resuspended at a concentration of 1 × 10^6^ cells/mL for flow cytometry assays using the LSRFortessa (BD Biosciences, New Jersey, USA), as previously established by our group [12]. The cell surface markers were prepared in 2 mL Eppendorf tubes, each containing a mix of specific monoclonal antibodies: CD45 FITC (Clone HI30—BD Biosciences, New Jersey, USA), Cytokeratin 14, 15, 16, and 19 PE (BD Biosciences, New Jersey, USA), EpCAM PerCP-Cy5.5 (Clone EBA1—BD Biosciences, New Jersey, USA), CD54 BV421 (Clone HA58—BD Biosciences, New Jersey, USA), HLA-DR APC (Clone L243—BD Biosciences, New Jersey, USA) and SARS-CoV-2 protein spike marker (anti-Spike—Sigma-Aldrich, Missouri, USA) stained with a secondary antibody (AF488—Abcam, Cambridge, UK). A 100 µL volume of the sample was added to the antibody mix and incubated for 30 min. For washing, 500 µL of 1 × PBS was added, followed by centrifugation at 3000 rpm and 18 °C for 7 min. After discarding the supernatant, a second wash was performed with 1 × PBS using the same procedure. The samples were then resuspended in 200 µL of 1% PFA (prepared in PBS) and transferred to cytometry tubes for immediate analysis on a Fortessa flow cytometer (BD Biosciences, San Jose, USA). The tubes were kept protected from light during the analysis. Unstained controls were added to each experiment. Gate strategy is presented in Figs. 3, 4, 5 and 7, whereas initial gating strategy is Time (Time x SSC-A) and Singlets (FSC-A and FSC-H) (data not shown).

### Evaluation of immune inflammatory mediators in tracheal aspirate samples from COVID-19 patients by luminex

To quantify the inflammatory mediators present in tracheal aspirate (TA) samples from COVID-19 patients, TA samples were initially cleared by centrifugation at 800 × g for 10 min at room temperature, and TA supernatants were filtered in 0.22 µm syringe filters and then transferred to fresh 2 ml microtubes, as described [13]. The samples were diluted 1:10 and incubated with magnetic beads coated with monoclonal antibodies specific for various immune mediators, such as chemokines (CXCL8, CCL3, and CXCL10), inflammatory cytokines (IL-1β, IL-6, TNF-α, TNF-β, IFN-γ, IFN-α, IL-15, IL-18, and IL-17α), regulatory cytokines (IL-1Ra, IL-9, IL-10, IL-13, and IL-22), and growth factors GM-CSF, IL-2, and IL-7). The experiments were performed according to the manufacturer’s instructions using the Procarta Human Cytokine 34-plex assay (Invitrogen, CA, USA) and the Bio-Plex Pro Human Cytokine 27-plex Assay (Bio-Rad, CA, USA). Immune mediators were measured in TA samples, and the concentrations of each sample were determined according to the standard curves run for each molecule tested using a fifth-parameter logistic fit analysis. Results were expressed in pg/mL for all mediators tested.

### Data analysis and statistics

Data analysis of flow cytometric data was performed using FlowJo v.10.1.8 (Becton Dickinson), while statistical analyses were carried out with GraphPad Prism 8.0 (GraphPad Software Inc.). Statistical comparisons between groups were conducted using ANOVA with Tukey’s post-test for parametric data and the Kruskal–Wallis test with Dunn’s post-test for non-parametric data. For two-group comparisons, Student’s t-test (parametric) or the Mann–Whitney test (non-parametric) was applied. Differences were considered statistically significant when *p* < 0.05. Correlation analysis was conducted using Pearson’s test for parametric data and Spearman’s test for non-parametric data. Significant “r” values ( ≥|0.67|) with *p* < 0.05 were used to construct comprehensive networks, highlighting only strong correlations.

## Results

**Fig. 1:**
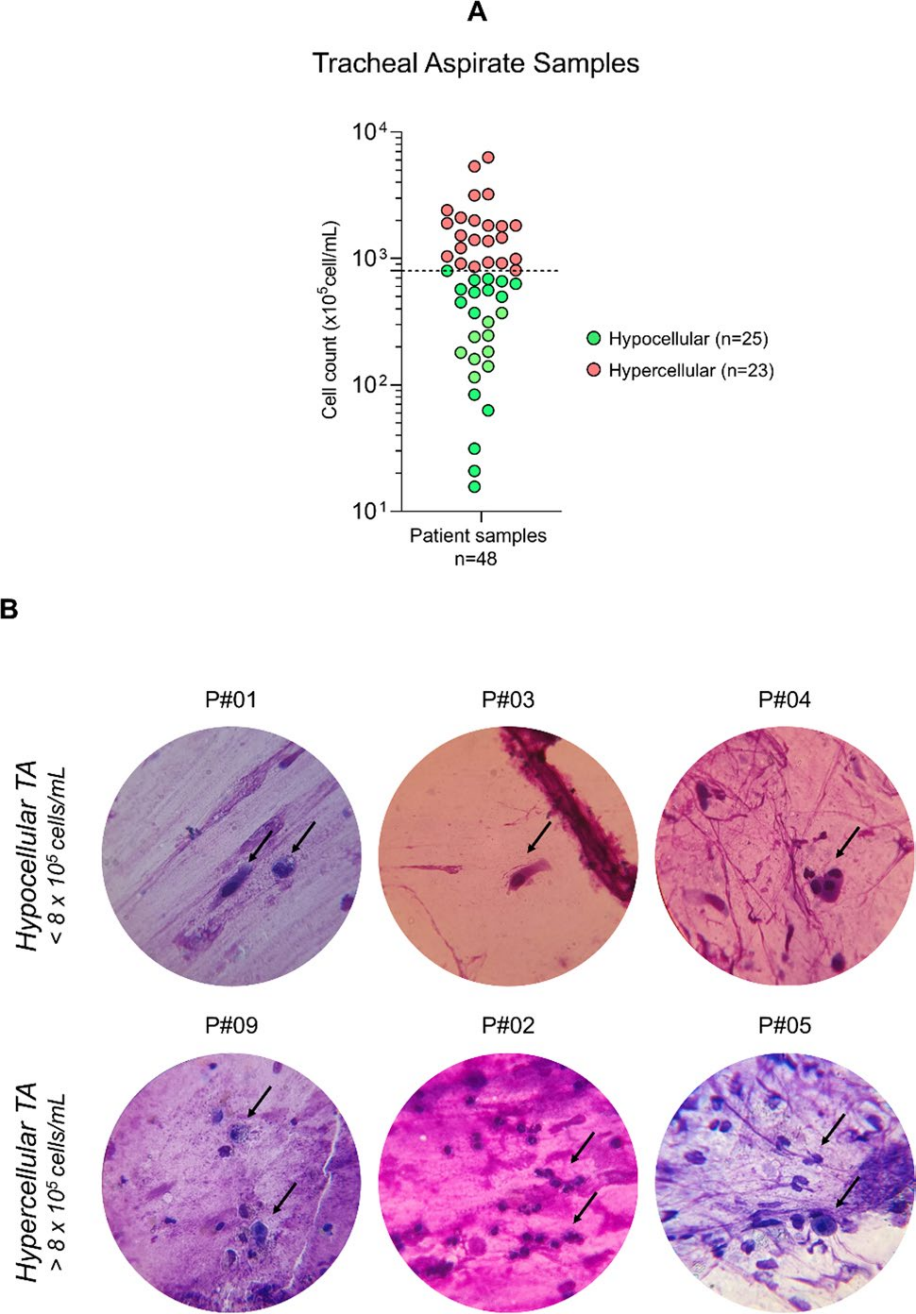
Classification of Tracheal Aspirate Samples from Severe COVID-19 Patients Based on Total Cell Count. **A** The X-axis represents the total number of patient samples (n = 48), while the Y-axis indicates the cell count relative to the global median (8 × 10^5^ total cells/mL). Each circle’s height corresponds to the cell count, as shown by the exponential numbers on the Y-axis. Red circles denote hypercellular samples (23 total), and green circles represent hypocellular samples (25 total). **B** Smear slides of TA samples from the COVID-19 patients. The six images represent patient samples stained with Panoptic rapid stain, with patient numbers assigned for reference. The arrows represent the different epithelial cells and inflammatory infiltrates found in the cellularity profiles. Images were captured at 100 × magnification

During data analysis, the biological samples were categorized into two distinct cellularity profiles based on the total number of cells per mL. An overall global median was calculated by evaluating the total cell count. Tracheal aspirate samples with up to 8 × 105 total cells/mL were classified as "hypocellular", while those exceeding the median were categorized as "hypercellular". Figure 1A illustrates the distribution of TA samples according to their cellularity. Following classification, morphological characteristics were evaluated using smear slides stained with Rapid Panoptic kit. Figure 1B showcases TA samples from six representative patients. Hypocellular samples exhibited fewer cells, while hypercellular samples showed a significantly higher number of cells, often with prominent inflammatory infiltrates. These morphological findings align with the cell count results. The smears also presented several morphological abnormalities, such as vacuoles and changes in shapes and sizes.

**Fig. 2:**
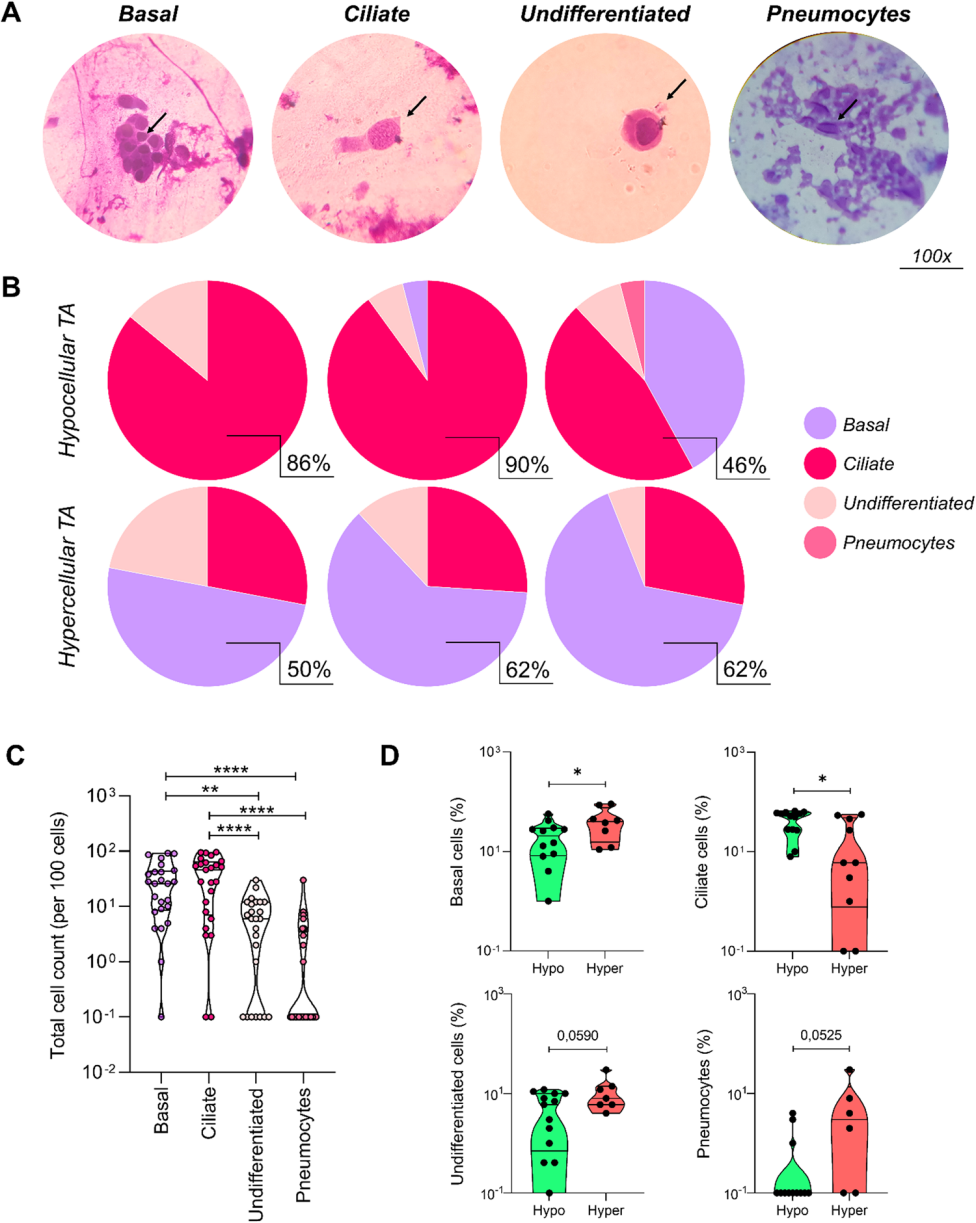
Cellular profile of tracheal aspirate samples from severe COVID-19 patients. **A** Representative images of the most observed cell types: basal, ciliated, undifferentiated, and pneumocytic cells, stained using Panoptic rapid staining, indicated by arrows. **B** Pie chart representation of the relative proportions of these cell types in TA samples classified as hypocellular and hypercellular profiles. **C** Overall predominance of basal and ciliated cells in the analyzed TA samples. **D** Comparative analysis of cell percentages between hypocellular and hypercellular profiles, highlighting statistically significant differences in basal and ciliated cells and trends in undifferentiated and pneumocyte cells. The results are presented as scatter plots showing individual data points overlaid with violin plots. Statistical analysis was conducted using the Kruskal–Wallis test followed by Dunn’s multiple comparisons test for group analysis, and the Mann–Whitney test for pairwise comparisons of non-parametric data. Significant differences (*p* < 0.05) are marked with an asterisk (*)

Microscopic analysis of TA smear slides revealed the most observed pulmonary epithelial cells during individual cell counts: basal, ciliated, undifferentiated, and pneumocytic cells, as shown in Fig. 2A. Basal cells displayed a dark purple cytoplasm and nucleus, while ciliated cells exhibited elongated light purple cytoplasm with cilia at the top and a slightly less stained nucleus at the base. Undifferentiated cells were characterized by a dark, purple-stained nucleus and a prominent light purple cytoplasm, and pneumocyte cells had an elongated dark purple nucleus, vacuoles, and light purple cytoplasm. Differential cell counts were conducted for each slide, with 100 cells per stained sample quantified into hypo- and hypercellular profiles. The results, illustrated in pie charts for six representative patients (Fig. 2B), highlighted a higher frequency of basal cells in hypercellular samples and an increase in ciliated cells in hypocellular samples. Overall, basal and ciliated cells predominated in TA samples from patients with COVID-19 (Fig. 2C), with pneumocyte cells being the least observed. Comparing the percentages of these four cell types in hypocellular and hypercellular samples revealed statistically higher values for basal cells in the hypercellular profile and ciliated cells in the hypocellular profile. Undifferentiated and pneumocyte cells showed a trend toward higher values in the hypercellular profile, though these differences were not statistically significant (Fig. 2D).

**Fig. 3:**
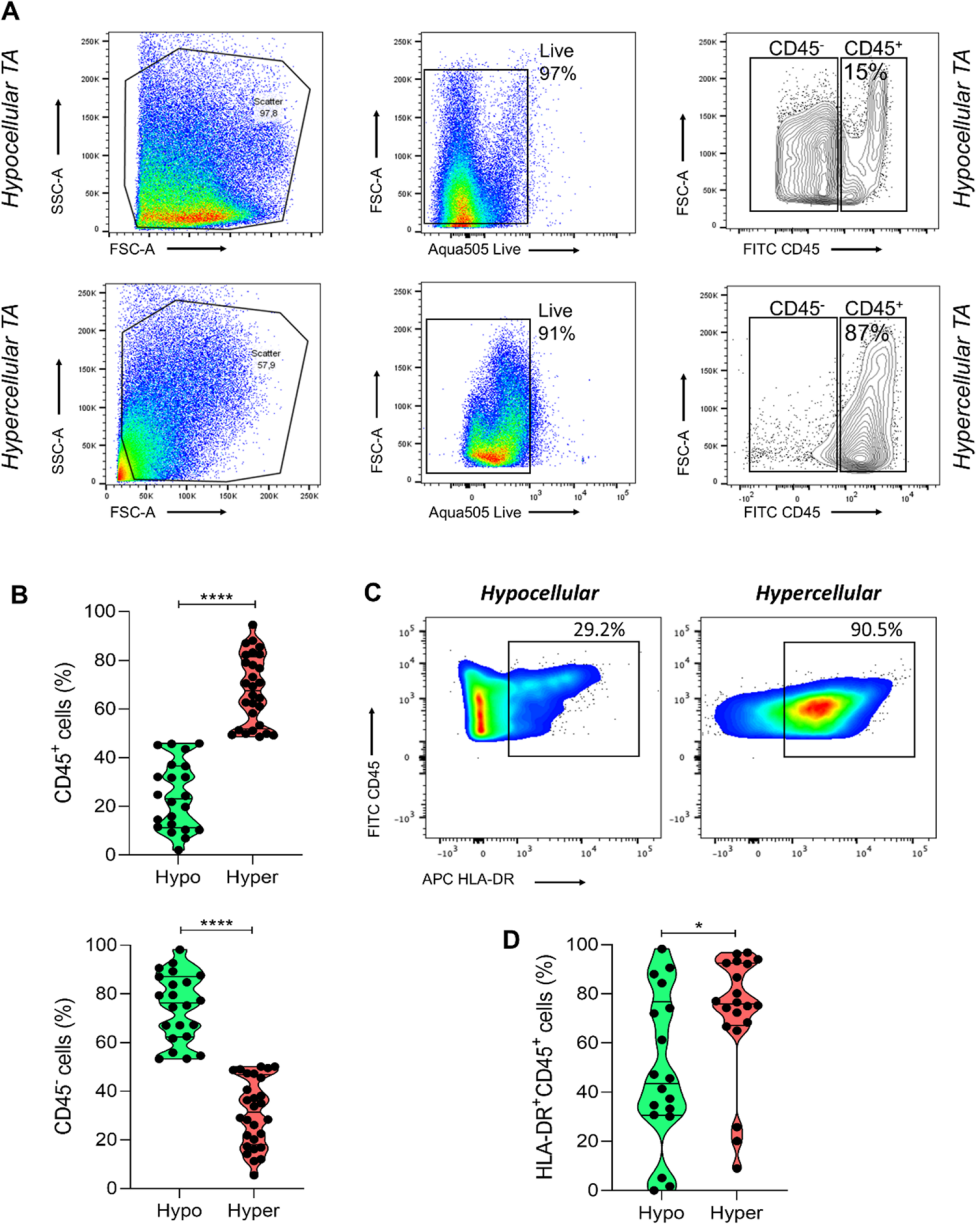
Distinct profiles of CD45 and HLA-DR in tracheal aspirate cells from patients with severe COVID-19*.* **A** Phenotypic analysis of tracheal aspirate samples by flow cytometry. Two-dimensional pseudocolor plots display SSC-A granularity versus FSC-A size for hypocellular (top) and hypercellular (bottom) profiles. Viable cells were identified using FSC-A versus Aqua505 staining. Within the viable cell gate, CD45 expression was assessed. **B** Percentage distribution of CD45^+^ (leukocytes) and CD45^−^ (epithelial cells) populations in hypocellular and hypercellular samples. **C** Pseudocolor plots showing CD45^+^HLA-DR^+^ cell percentages in hypocellular and hypercellular samples. **D** Quantification of CD45^+^HLA-DR^+^ cells across the two profiles. The results are presented as scatter plots showing individual data points overlaid with violin plots. Statistical analysis was conducted using the Mann–Whitney test for pairwise comparisons of non-parametric data. Significant differences (*p* < 0.05) are marked with an asterisk (*)

The TA samples were stained with various markers to evaluate the phenotypic and cellular activation by flow cytometry. This analysis revealed distinct leukocyte and epithelial cell frequency and patterns between hypocellular and hypercellular profiles, shown in Fig. 3A. Hypercellular samples showed a statistically higher concentration of CD45^+^ cells, indicating an abundance of leukocytes from the inflammatory infiltrate, whereas CD45^−^ cells, a cellular compartment of epithelial cells, were more frequent in hypocellular samples (Fig. 3B). Furthermore, the percentage of CD45^+^HLA-DR^+^ cells, a surrogate of leukocyte activation, were significantly higher in hypercellular samples as compared to hypocellular ones (Fig. 3C and D).

**Fig. 4:**
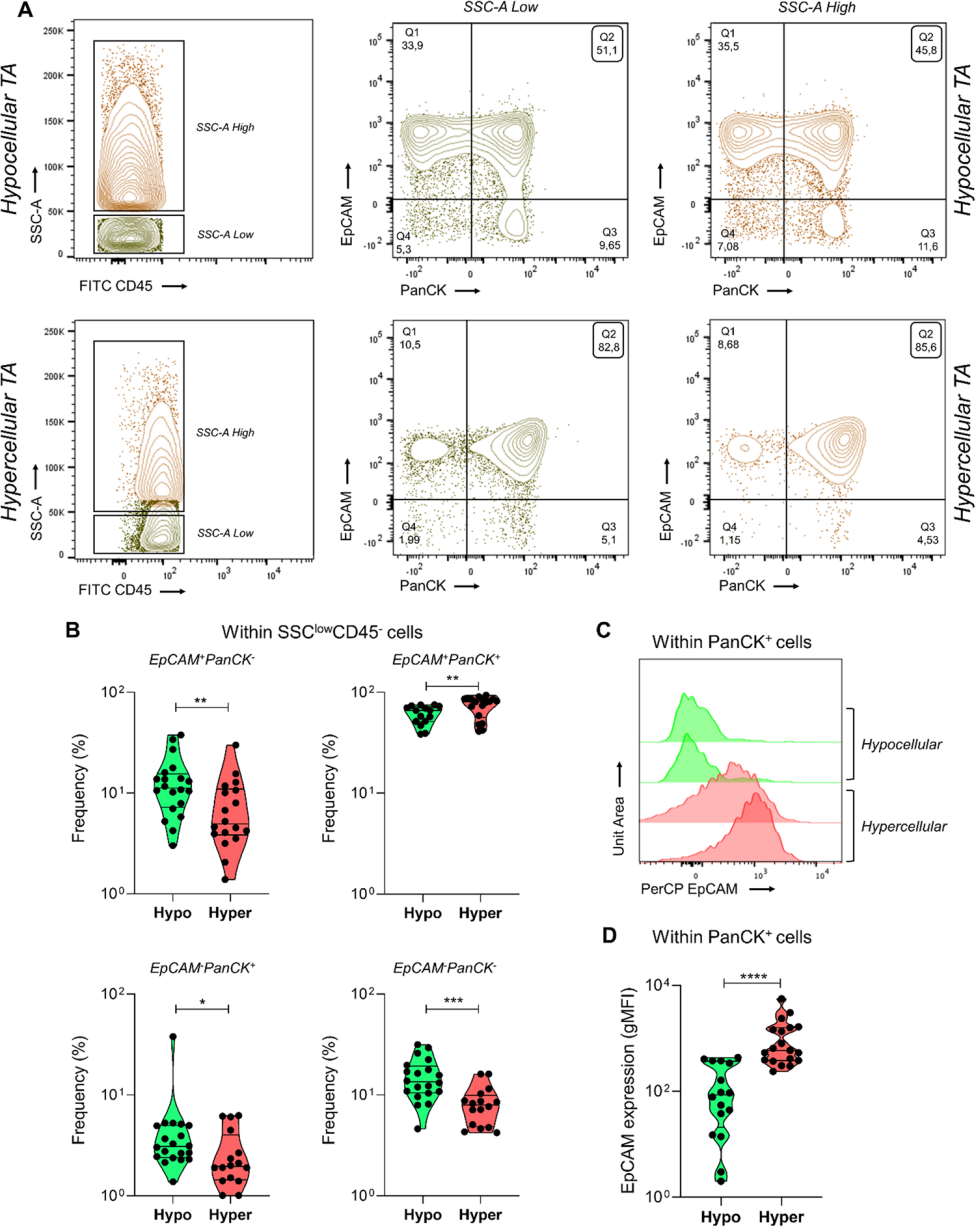
Epithelial cell marker EpCAM expression as a hallmark of cells from Tracheal Aspirate samples from COVID-19 patients*.* **A** Flow cytometry contour plots of CD45^−^ cells, showing the profile in hypocellular and hypercellular samples. High granularity is represented in orange and low granularity in green. Four quadrants were evaluated, Q1 = EpCAM^+^PanCK^−^, Q2 = EpCAM^+^PanCK^+^, Q3 = EpCAM^−^PanCK^+^ and Q4 = EpCAM^−^PanCK^−^. **B** Violin plot of percentage frequency of EpCAM and PanCK quadrants from hypocellular and hypercellular TA samples of SSC^low^CD45^−^ cells. **C** Flow cytometry histogram plot, with profiles of hypocellular (green) and hypercellular (red) samples demonstrating EpCAM expression. **D** Violin plot of hypocellular and hypercellular tracheal aspirate samples of CD45^−^ cells expressing EpCAM. Statistical analysis was conducted using the Mann–Whitney test for pairwise comparisons of non-parametric data. Significant differences (*p* < 0.05) are marked with an asterisk (*)

To further characterize these CD45^−^ cells, Cytokeratins 14, 15, 16, and 19 (hereafter referred to as PanCK) were analyzed to confirm the presence and frequency of epithelial cells in tracheal aspirate samples from patients with COVID-19. As shown in Fig. 4, the CD45^−^ cells were categorized into High Granularity (SSC-High) and Low Granularity (SSC-Low) gates to better define the diverse profile of the TA samples (Fig. 4A). The Epithelial Cell Adhesion Molecule (EpCAM) was also evaluated. EpCAM is a transmembrane glycoprotein prominently expressed on the surface of epithelial cells. The analysis of PanCK and EpCAM revealed four subpopulations of epithelial cells: EpCAM^+^PanCK^−^, EpCAM^+^PanCK^+^, EpCAM^−^PanCK^+^, and EpCAM^−^PanCK^−^ (Fig. 4A). Quantitative analysis showed that the EpCAM^+^PanCK^+^ double-positive phenotype is significantly increased in epithelial cells from patients with a hypercellular profile. In contrast, the other subpopulations were more prevalent in tracheal aspirates from patients with a hypocellular profile (Fig. 4B).

In a further analysis, PanCK^+^ cells exhibited higher EpCAM expression in tracheal aspirate samples from COVID-19 patients with a hypercellular profile. The histogram highlights CD45^−^ cells that are PanCK^+^, confirming the elevated EpCAM expression in tracheal aspirate samples from patients with COVID-19 (Fig. 4C and D).

**Fig. 5:**
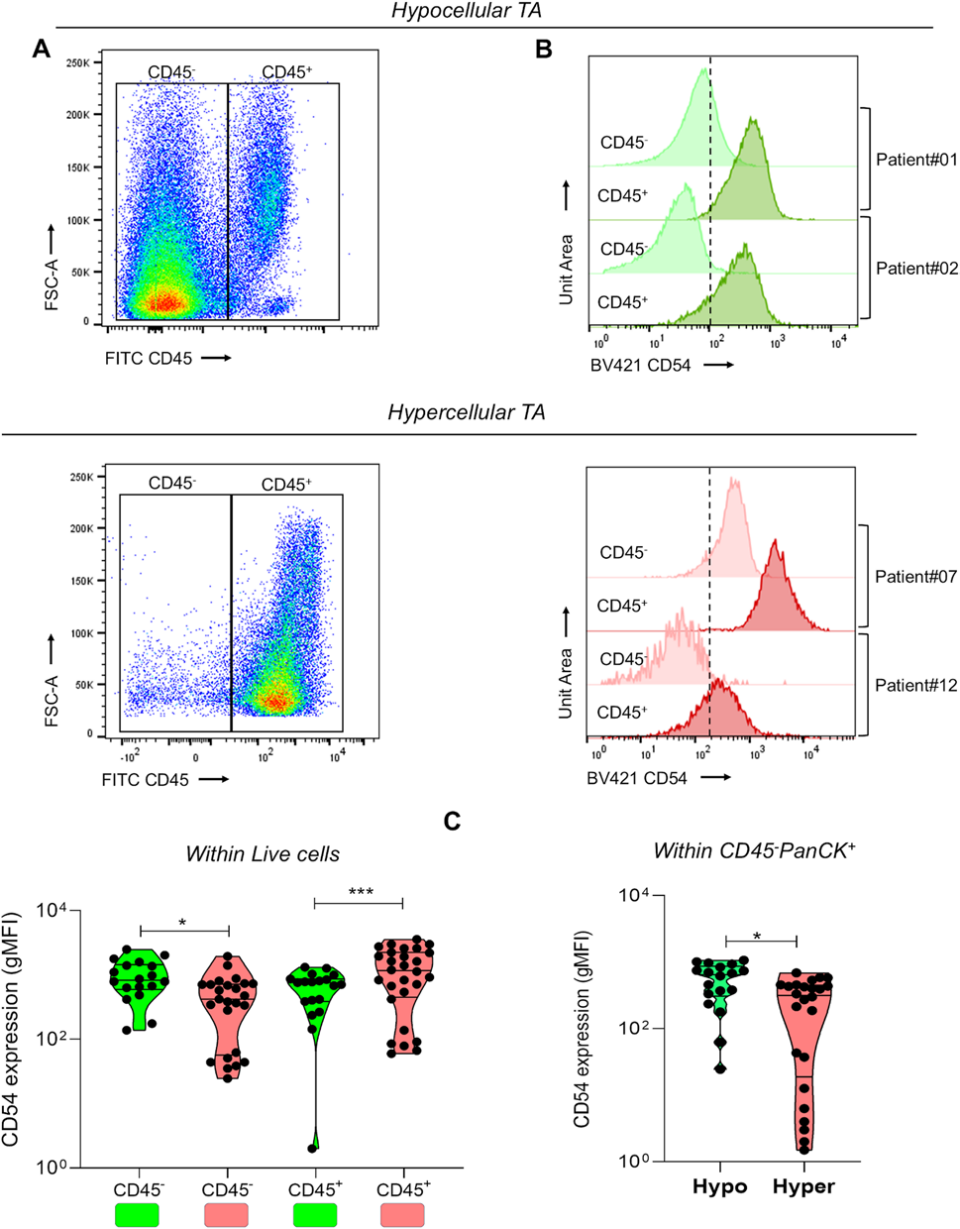
CD54 Adhesion molecule expression in tracheal aspirate samples from COVID-19 patients with a hypocellular and hypercellular profile. **A** Pseudocolor plots (left) display CD45^−^ and CD45^+^ cells, with cell size (FCS-A) characteristics represented on the Y-axis. **B** Histogram plots of CD54^−^ showing the geometric mean fluorescence intensity (gMFI) analysis in TA samples, separated into CD45^−^ and CD45^+^ cells. **C** Violin plots compare CD54 expression in hypocellular (green) and hypercellular (red) samples for both CD45^−^ and CD45^+^ cell populations. Statistical analysis was conducted using the Mann–Whitney test for pairwise comparisons of non-parametric data. Significant differences (*p* < 0.05) are marked with an asterisk (*)

For the purpose of investigating the activation of cellular components of the TA samples from COVID-19 patients, CD54 marker, an adhesion molecule associated with cell activation, was examined. The results demonstrated that CD45^+^ cells from TA samples with a hypercellular profile exhibit higher expression of the CD54 marker (Fig. 5A and B). Interestingly, in TA samples with a hypocellular profile, CD45^−^ cells display elevated CD54 expression, additionally within CD45^−^PanCK^+^ cells from hypocellular tracheal aspirates, higher CD54 expression is observed, confirming that epithelial cells in these samples express increased levels of this activation and adhesion marker (Fig. 5C).

**Fig. 6:**
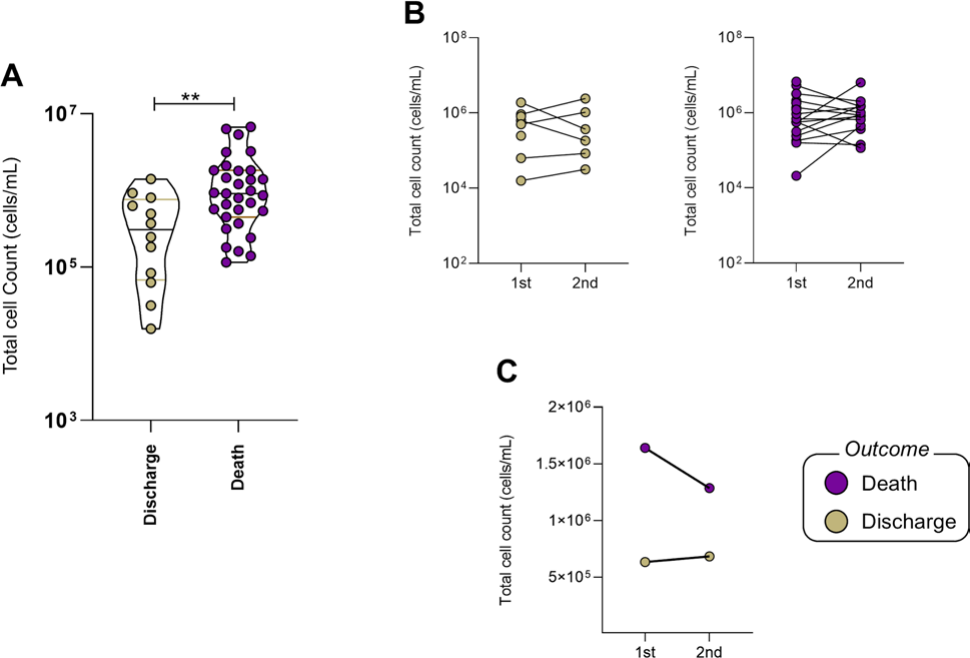
Analysis of the cellular profile and outcome of COVID-19 patients. **A** Violin plot overlaid with a scatter plot showing the cellularity of COVID-19 patient samples, categorized by outcomes of discharge (gray) and death (purple). **B** Total cell count (mL) in the first and second collections from patients with a discharge (left) and death (right) outcome. **C** Average cell count (mL) for the first and second collections, comparing outcomes of discharge (gray) and death (purple). Statistical analysis was conducted using the Mann–Whitney test for pairwise comparisons of non-parametric data. Significant differences (*p* < 0.05) are marked with an asterisk (*)

Intending to understand the association of the cellular profiles and death versus survival of COVID-19 patients, the analysis of cellularity and outcomes in COVID-19 patients, according to either discharge or death was performed. This analysis revealed that higher cellularity at ICU admission was found in individuals with a negative outcome (i.e., patients who progressed to death) (Fig. 6A). On the other hand, comparing the first TA collection (admission) with the second collection (7–14 days after admission), patients who progressed to death showed a decline in cellularity in the tracheal aspirate over time. In contrast, patients who progressed to discharge tended to maintain their cellularity levels. These findings suggest that a sustained hypocellular profile correlates with patients who recover and are discharged, resembling a system in homeostasis. Conversely, the hypercellular phenotype that declines over time is associated with patients who succumb to the disease, reflecting a profile linked to adverse outcomes associated with an early unsustained exacerbated inflammatory response and failure of the immune response (Fig. 6B and C).

**Fig. 7:**
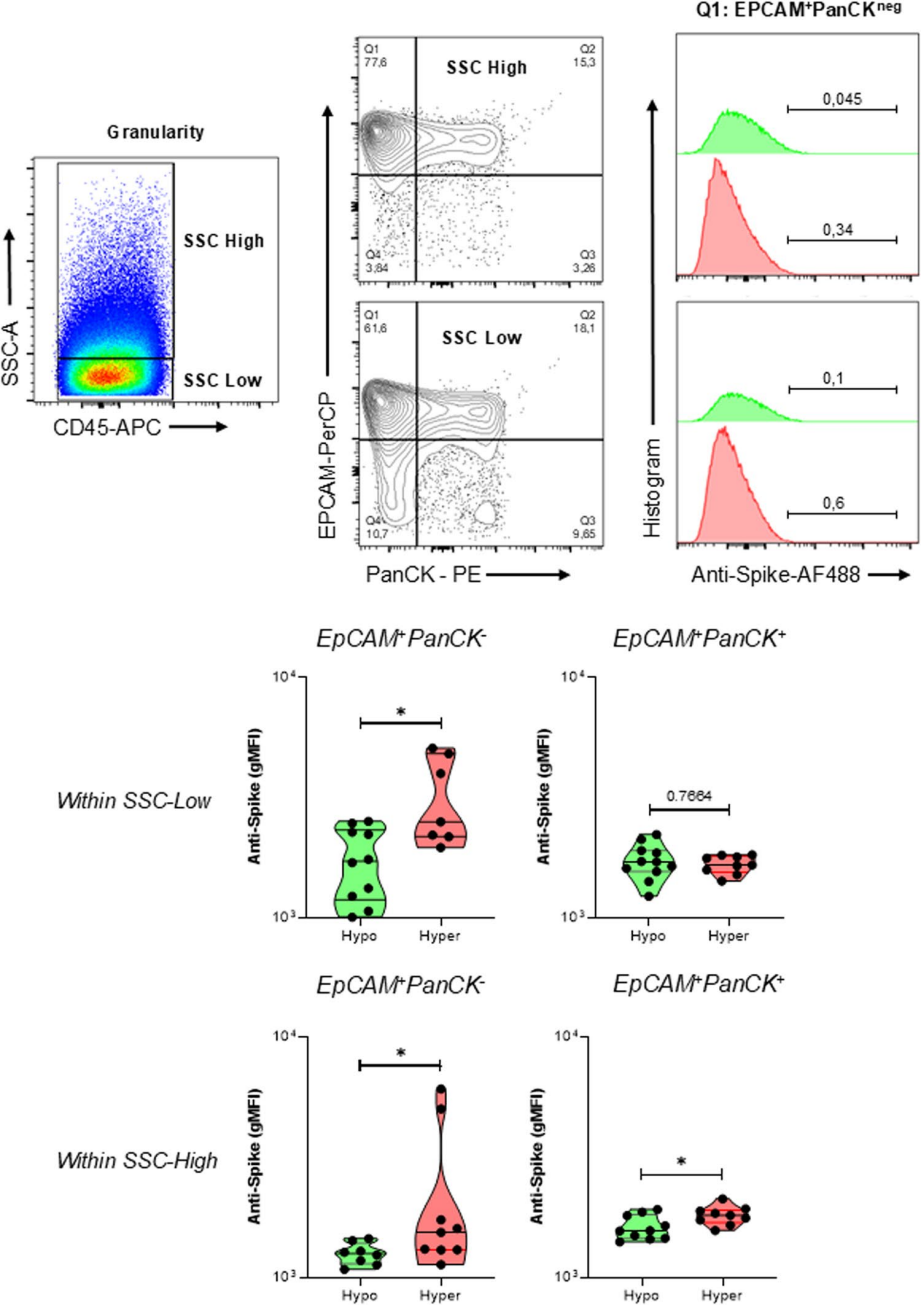
Expression of SARS-CoV-2 Spike protein in different subsets of EpCAM^+^cells from Tracheal Aspirate Samples of Patients with Severe COVID-19*.* **A** Pseudocolor plots (left) display anti-CD45 (APC) versus cell granularity (SSC-A) represented on the Y-axis. **B** Within high and low granularity, EpCAM and PanCK expressions were analyzed. Histogram plots of Spike-AF488 showing the geometric mean fluorescence intensity (gMFI) analysis in TA samples, separated into CD45^−^ and CD45^+^ cells were plotted. **C** Violin plots compare Spike expression in hypocellular (green) and hypercellular (red) samples for both CD45^−^ and CD45^+^ cell populations. Statistical analysis was conducted using the Mann–Whitney test for pairwise comparisons of non-parametric data. Significant differences (*p* < 0.05) are marked with an asterisk (*)

To further evaluate the presence of the SARS-CoV-2 in the hyper- and hypocellular samples, the tracheal aspirate samples were labeled with antibody anti-SARS-CoV-2 spike protein (anti-Spike-AF488). This new analysis demonstrated that the hypercellular samples had a higher expression of anti-Spike in EpCAM^+^ expressing cells regardless of PanCK expression, with no significant differences in solely PanCK^+^ or double negative cells (Fig. 7).

**Fig. 8:**
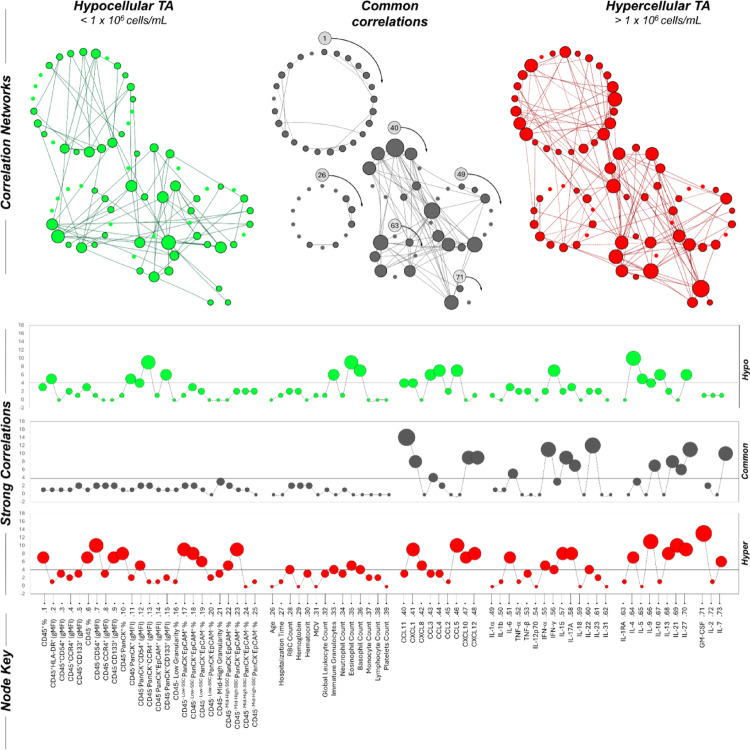
Interconnectivity analysis of serum soluble mediators, cellular, demographic/clinical and laboratorial attributes of hypocellular and hypercellular Tracheal Aspirate samples from severe COVID-19 patients*.* Networks were built based on correlation matrices, which were based on the Pearson and Spearman “r” scores among cellular, chemokines, pro-inflammatory, regulatory cytokines, growth factors and demographic/clinical and laboratorial attributes measured from COVID-19 patients. All data refers to admission TA samples. The number and position of each element is displayed in the legend above from 1 to 73, as clockwise arrows. Circular layouts were selected to display the significant correlations. Connecting lines represent strong correlations (“r” scores ≥|0.67|, thick gray lines) between pairs of elements, while negative correlations are underscored by dashed lines. The sizes of the nodes represent the number of connections made by each element, the more connections, the larger the node symbol. Signatures of the strong correlations was calculated by the number of correlation above the global median cutoff in each group

At last, the analysis of interconnectivity among the elements present tested during COVID-19 in tracheal aspirate samples was analyzed. In this analysis, soluble mediators were evaluated and their association of the subsets of TA samples was assessed. Data analysis as shown in Fig. 8 demonstrates the profile of networks composed by hypocellular and hypercellular TA samples. The results clearly demonstrate divergences with lack of connectivity of the cellular components with chemokines, cytokines and growth factors on the hypocellular TA as compared to hypercellular samples. Hypercellular TA networks showed abundant connectivity of CD54 adhesion molecule expression, as well as CD133. This molecule, which is a marker of epithelial cells and poor prognosis during cancer, was also described as a marker of cell hypoxia [14–16]. CD133 was highly correlated with other subsets as well as chemokines and proinflammatory cytokines. Regarding growth factors, GM-CSF demonstrated strong and numerous neighborhood connections, including with INF-α, other proinflammatory and regulatory factors as well as chemokines. Noteworthy was that hypocellular TA displays several negative connections, while hypercellular demonstrates a highly connected network with positive connections.

In addition, the strong correlations were separated into exclusive and common connections for each group, and again, it is possible to note the greater number of correlations between the cellular parameters of the hypercellular samples, especially in the compartments of cells expressing EpCAM. Moreover, among the inflammatory mediators there are a greater number of strong correlations within the common links, but the hypocellular profile shows a greater number of correlations between regulatory cytokines such as IL-4, IL-10 and IL-27. Overall, the correlation analysis displaying networks has corroborated the opposing profile of hypo and hypercellular TA profiles, highlighting the importance of the detailed scrutiny of these patterns for fully understanding COVID-19 in the respiratory tract in humans.

## Discussion

Our study is the first one that was able to identify two distinct profiles in TA samples from COVID-19, hypercellular and hypocellular, that were unique regarding cellular composition. Samples with hypercellularity had higher levels of basal cells, indicating that there is a greater involvement of the epithelial tissue in a more inflamed microenvironment. In that sense, the detachment of the basal layer can be derived from the direct cytopathic effect of viral replication but enhanced by the highly inflamed microenvironment observed in patients with severe COVID-19 [11, 17].

Considering that basal cells are lung stem cells, capable of differentiating into other cells of the airway epithelium such as ciliary and secretory cells, the exposure and loss of the basal cells can lead to the impairment of the renewal and repairment of the respiratory tract, allowing the establishment of permanent sequelae post SARS-CoV-2 infection [11, 18, 19].

Likewise, the increased presence of CD45^+^HLA-DR^+^ cells in patients with hypercellularity, reveals the strong presence of activated leukocytes in the tracheal aspirate composed mainly of activated neutrophils from the pulmonary infiltrate, also corroborating the idea of a robust pro-inflammatory pulmonary site for COVID-19 patients that has been amply described since the beginning of the pandemics [20, 21]. Neutrophil extracellular traps (NET) release by activated neutrophils induce fibrin deposition in the lungs, as can tissue factors produced by intra-alveolar neutrophils, further aggravating respiratory distress. Additionally, these cells are also important sources of proinflammatory cytokines, chemokines, proteases, reactive oxygen species and other molecules, exacerbating the tissue [11].

Comparable studies evaluating lower airway samples (particularly BALF and tracheal aspirates) in ARDS and infectious respiratory diseases support the concept that airway cellular composition reflects biologically meaningful heterogeneity rather than sampling variability. Previous studies have demonstrated that ARDS subtypes defined by etiology present differences in neutrophil, macrophage, and lymphocyte composition. For example, COVID-19–related ARDS has been associated with increased pulmonary T-lymphocyte presence compared with non-COVID ARDS, while infectious/pneumonia-related ARDS generally shows higher neutrophil and lymphocyte counts than non-infectious etiologies [22]. In addition, studies in critically ill COVID-19 patients have reported that BALF macrophage abundance correlates with clinical outcomes such as ICU mortality [23]. A previous study on lung injury during infant viral respiratory tract infection demonstrated that tracheal aspirates accurately reflect the airway environment while representing a minimally invasive sampling method integrated into routine clinical care [24]. These findings support the concept that variation in airway cellular composition reflects biologically meaningful differences.

Analysis of CD54 in leukocytes (CD45^+^) and epithelial cells (CD45^−^), pointed towards increased expression in CD45^+^ cells in hypercellular tracheal aspirate samples, whereas, CD45^−^PanCK^+^ cells in hypocellular samples have a higher expression of this marker when compared to samples with a hypercellular profile. In addition of being an endothelial cell adhesion molecule, CD54 marker was also found to be associated with antigen uptake in antigen presenting cells (APCs) [25], playing an important role for the interaction between phagocytes and T cells during immunological synapses in addition of inducing an effector phenotype of the T cell population [26].

In order to obtain a more detailed assessment of CD45^−^ cells, both the presence of Cytokeratins 14, 15, 16 and 19 (PanCK) and the Epithelial Cell Adhesion Molecule (EpCAM) were also evaluated. EpCAM is a transmembrane glycoprotein prominently expressed on the surface of epithelial cells. It plays multifaceted roles in regulating cell adhesion, proliferation, migration, and signaling [27]. Notably, EpCAM expressions are frequently altered in various pathological conditions, including inflammation and cancer, making it a valuable biomarker for analyzing epithelial cell populations [28]. As for PanCK, it is well known that cytokeratins are important not only to maintain cell integrity and cell division, but also to help with cellular repair following tissue damage, such as the one triggered by viral infections, in addition to forming a protective barrier against several pathogens such as viruses [29].

Our findings have shown not only an increase in double-positive cells (EpCAM^+^PanCK^+^) in patients with a hypercellular profile, but also an augmented expression of EpCAM^+^ molecules in epithelial cells in such samples. Studies have shown that EpCAM^+^ cells can produce cytokines and chemokines during infection with respiratory viruses, possibly aggravating the disease progression in fact, IL-6 production led by epithelial airway cells has been proposed as an initial hallmark of SARS-CoV-2 pathogenesis [30, 31]. Experimental studies have demonstrated that EpCAM signaling can interact with β-catenin activity, suggesting a functional relationship with the Wnt/β-catenin pathway, and Wnt signaling has been shown to regulate regenerative potential in EpCAM⁺ distal lung epithelial progenitor cells [32, 33]. That said, increased EpCAM expression may reflect epithelial remodeling or regenerative responses during severe lung injury. However, it should be emphasized that current mechanistic evidence originates mainly from non-human experimental models.

Furthermore, SARS-CoV-2 infection, identified by the expression of Spike protein, was significantly higher in EpCAM^+^ cells regardless of the granularity, and in EpCAM^+^PanCK^+^ cells with higher granularity, corroborating with these findings. Higher rates of infection in this compartment could be responsible for triggering inflammation and cytokine production, inducing leukocyte influx but with possible loss of airway epithelium/immune cells interplay dampening the effective immune response [34].

The detection of EpCAM^+^ circulating cells has already been proposed as a biomarker for pulmonary tumor progression during liquid biopsy being associated with worse prognosis [35]. Similarly, this study has shown that individuals with hypercellular profile presented higher death rates than individuals with hypocellular profile, therefore it is possible to assume that the occurrence of EpCAM^+^PanCK^+^ cells in the tracheal aspirate of COVID-19 patients could also be interpreted as a biomarker for negative outcomes.

Cellularity was the basis for defining the two dichotomic profiles of hypocellular and hypercellular samples. High cellularity profile has been a marker and strong indicator of inflammatory processes in human fluids, including bronchoalveolar liquid, liquor, and urine. In fact, we were unable to find studies that establish cellularity counts during COVID as a marker of disease progression. Therefore, in the present study we propose this parameter to distinguish between different patterns of inflammatory as well as epithelial cell profiles in tracheal aspirate samples from COVID patients.

In fact, a recent study from Shi and collaborators has provided a clinical dataset with publicly available records of cell counts and novel AI trained algorithms for detection and instance segmentation of bronchoalveolar lavage fluid (BALF) cells [36]. This recent study with robust sample size, the authors have demonstrated that the percentage of Neutrophils comprise 45% of BALF from patients with pulmonary disorders. The average for CD45 + Neutrophils in hypocellular samples was around 15% while it reached 85% when in hypercellular profile. These results corroborate our findings and our classification of samples in the hypo and hypercellular profiles.

This study has limitations that should be acknowledged. The sample size was limited due to the progressive reduction in eligible critically ill COVID-19 patients after widespread vaccination. Because tracheal aspirates are obtained exclusively from intubated individuals, establishing an appropriate non-COVID control group is inherently challenging, and direct comparisons with other causes of respiratory failure were not performed. Therefore, analyses focused on intra-cohort comparisons between hypo- and hypercellular profiles and clinical outcomes (discharge versus death). Importantly, the longitudinal design and repeated sampling in a subset of patients strengthened data robustness.

All in all, these results reveal unique local airway signatures of morbidity on critically ill COVID-19. It remains to be investigated if these signatures may correlate with or trigger later outcomes such as post-acute COVID sequelae and neoplastic conditions in convalescent patients.

## Supplementary Information

Below is the link to the electronic supplementary material.

Supplementary file1 (DOCX 19 KB)

## Data Availability

The results included in the present study are available from the corresponding author upon reasonable request.
