# Supplementary material for: SARS-CoV-2 drives upregulation of EpCAM in respiratory epithelial cells with hypercellular profile during severe COVID-19
Source: Inflamm Res. 2026 Jun 20;75(1):143. doi: 10.1007/s00011-026-02244-3 (PMC13283161; doi:10.1007/s00011-026-02244-3)
Supplement: Supplementary file 1 — Supplementary file1 (DOCX 19 KB) [file 11_2026_2244_MOESM1_ESM.docx]

**Supplementary Table 1 – Demographic and clinical characteristics of the COVID-19 patients**

|  | Outcome | |
| --- | --- | --- |
| Clinical Data | **Discharge** | **Death** |
| Number of patients (n) | 8 | 20 |
| Male (n) | 3 | 16 |
| Female (n) | 5 | 4 |
|  | Mean (±SD) | Mean (±SD) |
| Age (years) | 61.2 (±10.7) | 58.7 (±15.3) |
| Hospitalization (days) | 28.4 (±21.9) | 25.4 (±14.5) |
| Hematological Parameters |  |  |
| RBC (10^6^/µL) | 3.4 (±0.5) | 3.7 (±0.8) |
| Hgb (g/dL) | 10.1 (±1.6) | 11.1 (±2.9) |
| Ht (%) | 30.2 (±3.9) | 34.7 (±7.3) |
| VCM (µm^3^) | 88.9 (±8.3) | 93.24 (±9.5) |
| PLT (10^3^/µL) | 256.7 (±125.1) | 234.7 (±108.4) |
| WBC (10^3^/µL) | 12.5 (±4.3) | 14.3 (±9.8) |
| Band Neutrophils (10^3^/µL) | 0.279 (±0.8) | 0.094 (±0.3) |
| Segmented Neutrophils (10^3^/µL) | 12.186 (±11.9) | 12.157 (±9.04) |
| Eosinophils (10^3^/µL) | 0.126 (±0.13) | 0.269 (±0.5) |
| Basophils (10^3^/µL) | 0.068 (±0.12) | 0.013 (±0.05) |
| Monocytes (10^3^/µL) | 1.026 (±1.17) | 0.528 (±0.4) |
| Lymphocytes (10^3^/µL) | 1.367 (±0.957) | 1.245 (±0.822) |
| Gasometry |  |  |
| pH | 7.3 (±0.1) | 7.4 (±0.1) |
| pCO2 (mmHg) | 55.3 (±20.9) | 45.9 (±13.5) |
| pO2 (mmHg) | 105.2 (±31.7) | 98.8 (±40.3) |
| HCO3 (mmol/L) | 24.3 (±3.9) | 24.9 (±4.4) |
| SatO2 (%) | 95.4 (±6.6) | 93.4 (±8.96) |
| BE (mmol/L) | -3.4 (±3.1) | -0.7 (±4.8) |
| Potassium (K) (mmol/L) | 4.5 (±0.9) | 4.3 (±0.7) |
| Sodium (Na) (mmol/L) | 141.1 (±5.9) | 142.9 (±5.7) |
| Calcium (Ca) (mg/dL) | 4.96 (±0.3) | 4.9 (±0.7) |
| Cloride (Cl) (mmol/L) | 104.1 (±6.3) | 106.2 (±4.8) |
| Glucose (mg/dL) | 163.2 (±62.7) | 198.4 (±82.3) |
| Lactate (mmol/L) | 1.78 (±0.6) | 1.97 (±0.75) |
| Biochemical parameters |  |  |
| Creatinine (mg/dL) | 2.04 (±1.8) | 1.5 (±1.2) |
| Urea (mg/dL) | 91.26 (±65.3) | 99.27 (±64.8) |
